# Budd-Chiari Syndrome as an Initial Presentation of Essential Thrombocythemia

**DOI:** 10.7759/cureus.80700

**Published:** 2025-03-17

**Authors:** Ana Santos e Silva, Rodrigo Morgado, Monica Ferro Silva, Leonor Gama, Josiana Duarte

**Affiliations:** 1 Internal Medicine, Unidade Local de Saúde do Litoral Alentejano, Santiago do Cacém, PRT; 2 Internal Medicine, Hospital Do Litoral Alentejano, Santiago do Cacém, PRT; 3 Internal Medicine, Hospital Litoral Alentejano, Santiago do Cacém, PRT

**Keywords:** ascite, budd-chiari syndrome, essential thrombocythemia, jak 2 mutation, portal hypertension

## Abstract

Budd-Chiari syndrome (BCS) is a clinical condition defined by the obstruction of hepatic venous outflow, resulting in portal hypertension and subsequent hepatic dysfunction. The syndrome is frequently associated with prothrombotic states, among which myeloproliferative neoplasms represent the most prevalent underlying etiology.

The clinical presentation of BCS is highly variable, ranging from a mild and insidious disease course to an acute condition associated with liver failure. This heterogeneity in presentation often leads to delays in diagnosis.

This report describes a rare case of BCS as the first manifestation of essential thrombocythemia (ET) in a young male patient, emphasizing the importance of early diagnosis and multidisciplinary management for better outcomes.

## Introduction

Budd-Chiari syndrome (BCS) is defined as an obstruction of the hepatic venous outflow that may be due to obstruction of the hepatic veins or the inferior vena cava [1-7]. Presentation can be acute or insidious, and the condition is often underdiagnosed. Therefore, it’s important it remains a differential diagnosis in cases of portal hypertension of unknown etiology [4]. The most common primary cause is prothrombotic states, while secondary etiologies, such as tumor invasion and extrinsic compression of the hepatic vein, are rare, representing <1% of the cases [1, 4, 7, 8]. With thorough investigation, the underlying cause can be determined in over 80% of cases [2, 4]. Endovascular interventions and systemic anticoagulation have emerged as effective treatment strategies, leading to substantial improvements in patient outcomes [4, 7]. Liver transplantation is reserved for selected cases [4, 7].

Although myeloproliferative neoplasms, including essential thrombocythemia (ET), are common causes of BCS, this condition is a rare complication in patients with ET, accounting for 8% to 14% of BCS cases [4, 7, 9]. Essential thrombocythemia is a chronic myeloproliferative disorder characterized by an increased risk of both thrombotic and hemorrhagic phenomena and typically affects both the arterial and venous circulation [2, 8]. Budd-Chiari syndrome is a rare complication of ET [2, 9]. Treatment of ET primarily focuses on three areas: thrombotic risk mitigation, myeloproliferative management, and addressing complications [3].

## Case presentation

A 26-year-old male patient of Nepalese origin, employed in intensive agricultural work in Portugal for the past six months, was admitted to our hospital with complaints of abdominal distension and lower limb edema.

The patient had been asymptomatic until three months prior to admission, when he developed progressive fatigue, unintentional weight loss (approximately 6 kg), and later, abdominal distension and lower extremity edema, prompting medical evaluation. He had no fever, vomiting, changes in bowel habits, and no previous similar attacks.

At the time of admission, the patient was alert and oriented, with stable vital signs and a peripheral oxygen saturation of 98% on room air. Physical examination revealed decreased breath sounds in the left lung base, suggestive of pleural effusion, a positive fluid wave sign indicative of ascites, and bilateral pitting edema extending to the malleoli, consistent with fluid overload.

Laboratory studies (Table 1) on admission revealed evidence of coagulopathy, with an international normalized ratio (INR) of 1.82, a prothrombin time (PT) of 22.5 seconds (normal range: 9.0-13.0 seconds), and an activated partial thromboplastin time (aPTT) of 39.9 seconds (normal range: 23.8-35.8 seconds). Liver function tests showed hypoalbuminemia (3.4 g/dL (normal range: 3.5-5.2 g/dL)) and hyperbilirubinemia (total bilirubin 2.4 g/dL (normal range: 0.3-1.2 mg/dL), direct bilirubin 1.04 g/dL (normal range: 0.0-0.2 mg/dL)).

**Table 1:** Detailed laboratory evaluation on admission and serial measurements D: day; APTT: activated partial thromboplastin time; CRP: C-reactive protein; LDH: lactate dehydrogenase; AST: aspartate aminotransferase; ALT: alanine aminotransferase; GGT: gamma-glutamyl transferase; IGRA : interferon-gamma release assay; ADA: adenosine deaminase; Neg: negative

Date	D0	D1	D2	D5	D8	D20	D28	D32	Normal range
Total leukocyte count (/uL)	8400	10600	-	10900	9600	9000	5500	5400	4000 - 11000
Absolute neutrophil count (/uL)	6000	7400	-	8000	6600	5900	3300	3200	1600 - 8300
Absolute lymphocyte count (/uL)	1200	1700	-	1800	1800	2000	1400	1500	1300 - 3400
Hemoglobin (g/dL)	16.3	16.9	-	17.4	17.1	18.1	15.9	15.5	12.0 – 17.0
Platelets (/uL)	487000	485000	-	540000	538000	508000	498000	488000	150000 – 400000
Prothrombin time (seconds)	22.5	20.4	-	20.9	20.2	22.0	22.0	17.6	9.0 - 13.0
APTT (seconds)	39.9	38.7	-	38.1	36.8	40.1	40.8	40.0	23.8 – 35.8
CRP (mg/dL)	2.74	4.6	2.9	1.80	1.4	2.0	0.8	0.3	<0.5
Procalcitonin (ng/mL)	-	0.12	-	0.07	0.06	-	-	-	< 2.0
Urea (mg/dL)	25	21	19	27	17	18	17	18	<43
Creatinine (mg/dL)	0.9	0.9	0.8	0.9	0.8	0.9	0.8	0.8	0.8 – 1.2
Sodium (mEq/L)	134	140	-	138	136	140	140	141	136 – 146
Potassium (mEq/L)	4.3	3.9	-	4.1	4.2	3.9	4.0	3.7	3.5 – 5.1
Total protein (g/dL)	5.6	6.3	-	6.0	5.9	-	5.2	6.6	6.6 – 8.3
Albumine (g/dL)	3.4	3.8	-	3.5	3.5	-	3.0	4.1	3.5 – 5.2
LDH (UI/L)	334	189	154	187	225	200	154	122	208 – 378
AST (UI(/L)	55	62	48	60	66	73	54	44	0 – 50
ALT (UI/L)	50	55	41	48	48	47	35	33	0 - 50
GGT (UI/L)	-	97	86	103	107	123	104	102	0 - 55
Total bilirubin (mg/dL)	2.4	3.0	2.4	-	-	3.2	2.4	2.0	0.3 – 1.2
Direct bilirubin (mg/dL)	1.04	1.18	1.03	-	-	1.11	0.85	0.8	0-0.2
Alkaline phosphatase (UI/L)	67	76	63	79	87	105	92	92	30 - 120
IGRA	-	Neg		-	-	-	-	-	Neg
ADA (U/L)	-	-	7.9	-	-	-	-	-	0 - 15
Alpha-fetoprotein (ng/mL)	-	-	2.4	-	-	-	-	-	0 – 9

**Figure 1:**
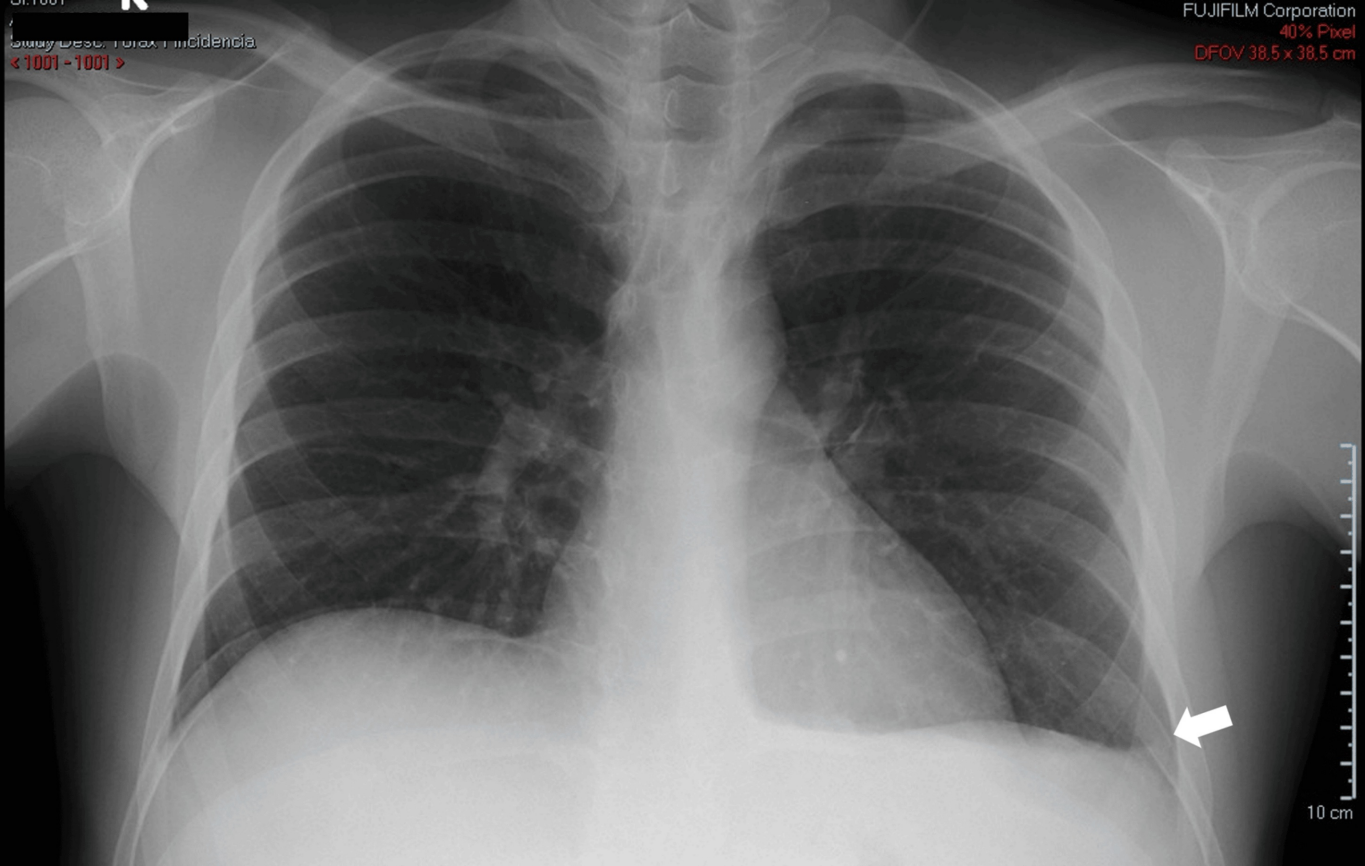
Chest X-ray showing blunting of the left costophrenic angle White arrow: left costophrenic angle

**Figure 2:**
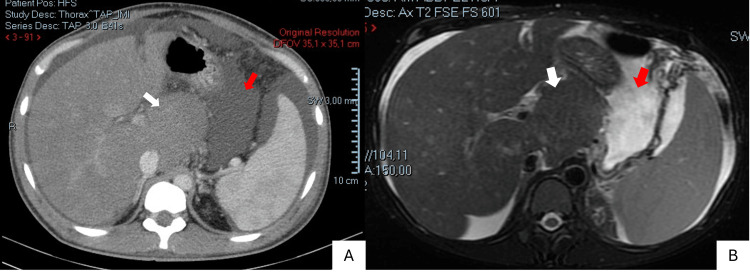
Abdominal and pelvic CT scan (A) and MRI (B) showing ascites and caudate lobe hypertrophy White arrow: caudate lobe hypertrophy; Red arrow: ascites

To further evaluate the patient's condition, a chest X-ray and a computed tomography (CT) scan of the abdomen and pelvis were performed. The chest X-ray revealed blunting of the left costophrenic angle, suggestive of a left-sided pleural effusion (Figure 1). The CT scan demonstrated hepatomegaly with diffusely homogeneous liver parenchyma, consistent with acute hepatitis. The caudate lobe was markedly hypertrophic (Figure 2). Additionally, large-volume ascites and thickening of the omental fat were noted. No masses or enlarged lymph nodes were identified.

During the emergency department stay, the patient developed a fever with a tympanic temperature of 38.6°C. Diagnostic paracentesis was performed, and given the findings (Table 2), empirical antibiotic therapy with ceftriaxone 2 g/day was initiated, assuming spontaneous bacterial peritonitis. Two blood cultures were also obtained. The patient was admitted to the hospital for further evaluation and treatment.

**Table 2:** Laboratory findings of ascitic fluid LDH: lactate dehydrogenase; ADA: adenosine deaminase; Neg: negative

Ascitic fluid		Value	Normal range
Macroscopic	Macroscopic examination	Hemorrhagic	-
Differential cell count	Leukocytes (/mm^3^)	488	<500
	Polymorphonuclear (%)	68	<50%
	Mononuclear (%)	32	≥25%
Biochemical parameters	Glucose (mg/dL)	124	>60
	Amylase (U/L)	42	27-131
	LDH (UI/L)	79	<200
	Albumin (g/dL)	1.2	<2,5
	ADA (U/L)	2.7	<40
Microbiological study	Gram stain	Neg	Neg
	Bacterial culture (aerobic + anaerobic)	Neg	Neg
	Ziehl-Neelsen stain – Acid-fast bacteria	Neg	Neg
	*Mycobacterium *culture (Lowenstein-Jensen)	Neg	Neg

Regarding spontaneous bacterial peritonitis, the patient's clinical condition improved significantly within 24 hours of initiating antibiotic therapy with resolution of the fever spikes and inflammatory markers returning to normal levels.

**Figure 3:**
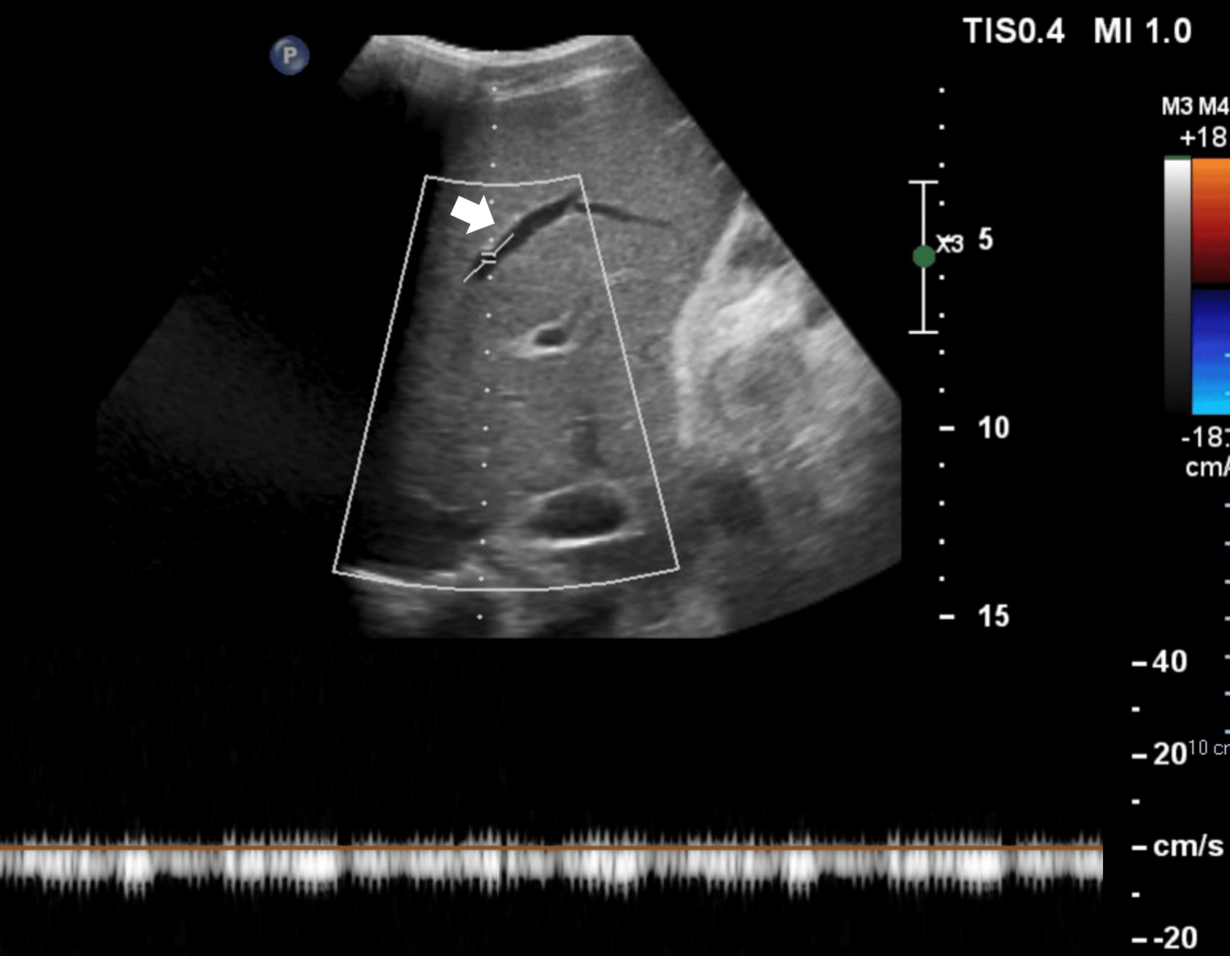
Abdominal ultrasound with Doppler demonstrating loss of the normal respiratory variability of the hepatic veins. White arrow: middle hepatic vein

To further investigate the patient's condition, an abdominal ultrasound with Doppler was performed. This revealed loss of normal respiratory variation in the hepatic and suprahepatic veins, suggestive of hepatic venous outflow obstruction (Figure 3). Subsequent abdominal magnetic resonance imaging (MRI) confirmed these findings, demonstrating hepatomegaly with a markedly enlarged caudate lobe, absent hepatic veins, and a narrowed portal vein with evidence of intraluminal thrombosis (Figure 2). Additionally, the imaging revealed significant ascites and a large left pleural effusion. Based on these findings, a diagnosis of BCS was made.

Immediately after the diagnosis, hypocoagulation with enoxaparin was initiated. Considering the clear improvement with medical therapy, the multidisciplinary team opted to defer endovascular intervention.

To investigate potential hypercoagulable states, genetic testing was performed, revealing the presence of the V617F mutation in the JAK2 gene. Subsequently, a bone marrow biopsy was performed, revealing a normocellular bone marrow with a normal myeloid/erythroid ratio. There was an evident proliferation of megakaryocytes with hyperlobulated nuclei and normal morphology of the granulocytic and erythroid lineages. Next-generation sequencing (NGS) was also conducted, identifying a missense mutation in exon 14 of the JAK2 gene (9p24.1). Based on these findings, a diagnosis of ET was established.

The patient was initiated on pegylated interferon alfa-2a therapy under the guidance of the hematology department and discharged with a multidisciplinary follow-up plan involving internal medicine, hematology, and hepatobiliary surgery.

## Discussion

Budd-Chiari syndrome is the result of an obstruction of suprahepatic venous outflow, anywhere from the liver to the heart, leading to hepatic congestion, thus increasing portal vein pressure with the risk of progression to hepatic fibrosis or liver failure [1-7]. The clinical presentation of BCS is extremely variable and can range from an accidental finding to fulminant liver failure [1,3,7].

The epidemiology of BCS is poorly defined, with studies reporting an annual incidence of 0.168 to 4.09 per million and a prevalence of 2.40 to 33.10 per million [6-8]. Budd-Chiari syndrome can occur at any age but is most prevalent between the ages of 19 and 49 years, with a median age of 35-40 years [4,6]. While it predominantly affects females in Europe and the United States, males are slightly more likely to be affected in India and Japan [4,6].

The clinical manifestations of BCS vary widely, ranging from asymptomatic cases to severe presentations with abdominal pain, ascites, hepatomegaly, and signs of portal hypertension [4-7]. In chronic cases, the development of regenerative nodules can lead to hepatic fibrosis and portal hypertension, increasing the risk of hepatocellular carcinoma [4-7]. However, in approximately 10% of cases, the condition may be asymptomatic [4-6]. The diversity of clinical presentations is not solely attributed to the acute or chronic nature of the obstruction but also to the specific vessel involved [4,7]. Obstruction of the hepatic veins is more commonly associated with acute conditions, whereas inferior vena cava obstruction tends to present more chronically [4]. The geographic disparities in the presentation of this condition are also notable, with patients from low-resource countries often experiencing a more gradual onset of symptoms and a lower association with prothrombotic disorders [4, 6, 7, 10]. In contrast to the findings from recent studies in Nepal, our patient did not exhibit preferential involvement of the inferior vena cava [4].

An underlying disease, hereditary or acquired hypercoagulable state, is identified in approximately 75% of BCS patients, with one-third of them exhibiting at least one prothrombotic condition [6,7]. The most prevalent prothrombotic factors in BCS are myeloproliferative disorders (40% to 60% of patients), antiphospholipid syndrome (10% to 12%), and paroxysmal nocturnal hemoglobinuria (7% to12%) [6, 7, 11]. The pathogenesis of myeloproliferative disorders-associated splanchnic veins thrombosis remains unclear [11]. However, endothelial cells harboring the JAK2 V617F mutation have been identified in liver endothelial cells in BCS and circulating endothelial progenitor cells, suggesting a potential role in disease development [11]. Leyden factor V mutations, deficits in proteins C and S, and hormonal factors have a prevalence between 1% and 8% of the patients [6]. Polycythemia vera is responsible for around 10% to 43% of cases of this syndrome and ET for 8% to 14% [2,4,11]. Given the prevalence of underlying hematological disorders, it is essential to investigate somatic mutations in the JAK2, including V617F and exon 12 mutations, as well as mutations in the CALR and MPL genes [6,11]. Additionally, an evaluation of hereditary deficiencies in proteins C, S, and antithrombin should be performed [6].

Budd-Chiari syndrome is frequently underdiagnosed, with a median diagnostic delay of approximately six months [4]. The diagnosis of BCS is confirmed by evidence of hepatic flow obstruction [1, 5, 6]. Despite several studies, the heterogeneity between them hinders the definition of the ideal method for diagnosis [3-6]. Abdominal Doppler ultrasound is an effective diagnostic method of BCS, with a sensitivity and specificity of 85%, also allowing the assessment of the hemodynamic impact of the findings, making it a good first-line choice [3-6]. However, operator dependence often leads to the decision to complement the findings with other imaging modalities, such as CT or MRI [1,3,5,6]. Computed tomography imaging enables precise evaluation of the location and degree of venous stenosis or obstruction, providing essential anatomical information for surgical planning [4-6]. An MRI is helpful in defining the extent of liver parenchymal involvement and characterizing associated abnormalities, such as nodular lesions [4-6]. If these studies remain inconclusive, a liver biopsy should be considered, as a histological examination is crucial in cases of BCS involving small hepatic veins [4,6,10].

The imaging manifestations of BCS are stage-dependent, with acute, subacute, and chronic phases exhibiting different characteristic imaging features [4-6]. The presence of hepatomegaly, heterogeneous liver echotexture, caudate lobe hypertrophy, and ascites in our patient is indicative of an acute phase of the disease [4,6]. To fully assess patients with BCS, imaging confirmation should be complemented by a comprehensive evaluation of portal hypertension complications, including analysis of ascitic fluid, exclusion of esophageal varices by upper endoscopy, and assessment of coagulation function [4,6].

Despite the development of several prognostic scores for BCS, their clinical utility in patient management remains limited [4, 6].

The treatment of BCS consists of two main components: anticoagulation and hepatic venous decompression, which can be performed either endovascularly or surgically [1, 2, 4-7]. Lifelong anticoagulation is recommended, even in the absence of identifiable prothrombotic disorders, and typically initiated with unfractionated or low-molecular-weight heparin, followed by transition to warfarin or direct oral anticoagulants (DOACs) [4, 6, 7]. While the efficacy and safety of warfarin and DOACs seem comparable, the lack of a specific antidote for some DOACs may raise concerns in patients with a higher bleeding risk [4, 7]. Additionally, addressing the underlying cause of BCS is crucial [4, 6, 7, 9].

Endovascular therapy aims to relieve hepatic venous outflow obstruction and may be achieved through angioplasty, endovascular shunts, or thrombolysis [4, 6, 7]. Angioplasty, with or without stent placement, is currently the preferred treatment modality and is performed in 40% to 93% of patients [4, 7]. Stenting is generally indicated in cases of inadequate pressure relief or recoil after angioplasty [4, 7]. Transjugular intrahepatic portosystemic shunt (TIPS) is reserved for patients who are ineligible for angioplasty or who experience persistent symptoms after angioplasty [4, 6, 7]. Although some cases of successful thrombolysis in acute BCS have been reported, it is not a commonly used approach [4,7]. Liver transplantation is reserved for patients who have failed other treatments or are not suitable candidates for alternative therapies [2, 6, 7]. It may also be considered for patients presenting with acute liver failure [6, 7].

The prognosis of BCS has improved significantly in recent decades due to advances in diagnostic imaging, anticoagulation therapy, and endovascular interventions, with five-year survival rates now exceeding 75% [4, 6]. In-hospital mortality is estimated at 4.9%, with increased risk in males, older adults, and those with cardiopulmonary or metabolic hematological comorbidities and hematological malignancies [7]. The five-year survival rate for patients undergoing endovascular therapy is 76.4%, with a higher survival rate of 88.6% observed in patients treated with angioplasty compared to 72.1% in those requiring TIPS [4, 6].

Myeloproliferative neoplasms arise from the clonal expansion of hematopoietic stem cells, resulting in excessive production of mature blood cells [7]. The mechanism by which myeloproliferative diseases lead to hypercoagulability is resistance to activated protein C and a reduction in free protein S levels leading to an increased activation of blood and endothelial cells [7, 9]. Patients with myeloproliferative diseases and BCS are typically younger, female, with inherited thrombophilia, and with the JAK2 V617F mutation [7]. In the case of our patient, the diagnosis of ET was established.

Essential thrombocythemia is frequently identified incidentally, with routine blood tests revealing an elevated platelet count; thrombocytosis is defined as a platelet count > 450,000 platelets per cubic millimeter [2, 11-13]. Essential thrombocythemia is characterized by thrombocytosis, megakaryocytic hyperplasia, and a clonal marker [9]. Patients often remain asymptomatic until the development of disease-related complications [2, 9, 11, 12]. Thrombotic events in ET can be either arterial or venous, with venous thrombosis occurring prior to diagnosis in approximately 8% of cases [9, 12, 13].

The annual incidence of ET is estimated at 1.2 to 3.0 cases per 100,000 individuals [9, 12]. The median age at diagnosis is 58 years, and there is a female prevalence (67% of patients are females) [12].

Diagnosing ET poses a challenge, requiring the exclusion of other myeloproliferative neoplasms and reactive thrombocytosis [2, 9, 12]. The presence of V1617F mutations in the JAK2 gene, CALR, or MPL mutations in association with thrombocytosis, normal hemoglobin, and serum lactate dehydrogenase levels, and the absence of leukoerythroblastosis or dacryocytes is highly suggestive of ET, but definitive diagnosis requires a biopsy of the bone marrow [9, 11, 12]. Notably, ET may present as triple-negative, lacking the JAK2 V617F, CALR, and MPL mutations in 10% to 20% of cases [9, 11, 12]. Given our patient's presentation meets all four major WHO diagnostic criteria for ET (Table 3) [13], the diagnosis of ET can be confidently established [8, 11, 12].

**Table 3:** WHO diagnostic criteria for essential thrombocythemia Source: [13]

WHO diagnostic criteria for essential thrombocythemia	
Diagnosis requires all four major criteria or the first three criteria plus a minor criteria	
Major criteria	1. Platelet count ≥450,000 per cubic millimeter; 2. Bone marrow biopsy showing proliferation mainly of the megakaryocytic lineage, with increased numbers of enlarged, mature megakaryocytes with hyperlobulated nuclei. No significant increase or left shift in neutrophil granulopoiesis or erythropoiesis; in rare instances, minor (grade 1) increase in reticulin fibers; 3. Criteria for BCR-ABL1–positive chronic myeloid leukemia, polycythemia vera, primary myelofibrosis, or other myeloid neoplasm not met; 4. JAK2 V617F, CALR, or MPL mutation
Minor criteria	1. Presence of clonal marker or of evidence of reactive thrombocytosis; 2. Absence of criteria for reactive thrombocytosis

While ET treatment does not substantially affect overall survival or the risk of leukemic transformation or myelofibrosis, it plays a crucial role in reducing the risk of thrombotic events [9, 11-13]. Treatment strategies should be individualized based on each patient's specific thrombotic risk profile (Table 4) [9, 11, 12, 14, 15].

**Table 4:** IPSET: thrombosis risk for essential thrombocythemia ^1^: one or more of hypertension, diabetes mellitus, or active smoking; IPSET: International Prognostic Score for Essential Thrombocythemia Source: [15]

IPSET: thrombosis risk for essential thrombocythemia	
Age under 60 years	0
60 years or older	1
Prior thrombotic event	2
Cardiovascular risk factors present ^1^	1
Detected JAK2 V617F mutation	2

In very low-risk patients, a watchful waiting approach may be appropriate [9, 12]. In higher-risk patients, low-dose aspirin may be the initial treatment, especially for those with the JAK2 V617F mutation, but existing guidelines are not consensual [9, 11, 12]. In high-risk patients (previous vascular event or V617F JAK2 mutation in patients over 60 years of age), hydroxyurea is recommended as initial therapy [9, 11, 12, 14]. Due to the potential for gonadal toxicity, mutagenicity, and teratogenicity, hydroxyurea is generally avoided in patients under the age of 40 [9, 12]. In these cases, pegylated interferon alfa-2a or busulfan may be considered [9, 12]. Pegylated interferon alfa-2a was started in our patient, the main advantages of which are the absence of genotoxicity or leukemogenic effects, higher rates of complete hematological response, and anti-clonal activity, with a minority of patients even achieving a complete molecular response [12]. The optimal target platelet for platelet count in ET patients on therapy remains uncertain [11, 12, 14]. Due to side effects, achieving a normal platelet count may not be feasible, leading to maintenance therapy with the maximum tolerated dose [12]. Age is the primary determinant of survival in ET, with older patients having a significantly shorter median survival (8.1 years) compared to younger patients (34.7 years) [12].

Our patient presented at a young age with a thrombotic complication of ET presenting with BCS. Despite the typical association of these patients with the V617F mutation of the JAK2 gene, the particular difficulty in diagnosis of ET in this syndrome stands out, since the elevated platelet count characteristic of ET might be concealed by factors such as portal hypertension and hypersplenism [2]. Given the patient's age and history of thrombosis, pegylated interferon alfa-2a was initiated. Long-term prognosis depends on ET progression, liver effects of BCS, and the risk of progression to chronic liver disease. Therefore, ongoing multidisciplinary monitoring is crucial to optimize patient care.

## Conclusions

Budd-Chiari syndrome is a rare condition associated with a significant risk of progression to hepatic fibrosis and liver failure. Early diagnosis and prompt initiation of targeted therapy are crucial to mitigating further damage to the hepatic parenchyma, thereby reducing the risk of fibrosis progression and subsequent liver failure. Consequently, this syndrome should always be considered in cases of portal hypertension with unclear etiology.

Given the high prevalence of hypercoagulable states among these patients, a thorough investigation of underlying prothrombotic conditions is essential, as BCS often represents the initial manifestation of other systemic disorders. Notably, the timely identification of these conditions, particularly myeloproliferative neoplasms, has a significant impact on both the therapeutic approach and overall prognosis.

## References

[1] Iliescu L, Toma L, Mercan-Stanciu A, Grumeza M, Dodot M, Isac T, Ioanitescu S (2019). Budd-Chiari syndrome - various etiologies and imagistic findings. A pictorial review. Med Ultrason.

[2] Coban S, Ertugrul I, Ekiz F, Akif Teber M, Yuksel O (2010). Budd-Chiari syndrome and portal vein thrombosis due to essential thrombocytosis. Platelets.

[3] Li Y, De Stefano V, Li H, Zheng K, Bai Z, Guo X, Qi X (2019). Epidemiology of Budd-Chiari syndrome: a systematic review and meta-analysis. Clin Res Hepatol Gastroenterol.

[4] Sharma A, Keshava SN, Eapen A, Elias E, Eapen CE (2021). An update on the management of Budd-Chiari syndrome. Dig Dis Sci.

[5] Gupta P, Bansal V, Kumar-M P (2020). Diagnostic accuracy of Doppler ultrasound, CT and MRI in Budd Chiari syndrome: systematic review and meta-analysis. Br J Radiol.

[6] Garcia-Pagán JC, Valla DC (2023). Primary Budd-Chiari syndrome. N Engl J Med.

[7] Haque LY, Lim JK (2020). Budd-Chiari syndrome: an uncommon cause of chronic liver disease that cannot be missed. Clin Liver Dis.

[8] Tefferi A, Barbui T (2020). Polycythemia vera and essential thrombocythemia: 2021 update on diagnosis, risk-stratification and management. Am J Hematol.

[9] Koschmieder S (2020). How I manage thrombotic/thromboembolic complications in myeloproliferative neoplasms. Hamostaseologie.

[10] Mancuso A (2020). Agreed diagnostic criteria needed for Budd-Chiari syndrome. Br J Radiol.

[11] Guglielmelli P, Vannucchi AM (2020). Current management strategies for polycythemia vera and essential thrombocythemia. Blood Rev.

[12] Tefferi A, Pardanani A (2019). Essential thrombocythemia. N Engl J Med.

[13] Arber DA, Orazi A, Hasserjian R (2016). The 2016 revision to the World Health Organization classification of myeloid neoplasms and acute leukemia. Blood.

[14] Galvez C, Stein BL (2020). Thrombocytosis and thrombosis: is there really a correlation?. Curr Hematol Malig Rep.

[15] Barbui T, Finazzi G, Carobbio A (2012). Development and validation of an International Prognostic Score of thrombosis in World Health Organization-essential thrombocythemia (IPSET-thrombosis). Blood.

